# Novel Liver X Receptor Ligand GAC0001E5 Disrupts Glutamine Metabolism and Induces Oxidative Stress in Pancreatic Cancer Cells

**DOI:** 10.3390/ijms21249622

**Published:** 2020-12-17

**Authors:** Shivangi Srivastava, Scott Widmann, Charles Ho, Donovan Nguyen, Alexis Nguyen, Asitha Premaratne, Jan-Åke Gustafsson, Chin-Yo Lin

**Affiliations:** Center for Nuclear Receptors and Cell Signaling, Department of Biology and Biochemistry, University of Houston, Houston, TX 77204, USA; shivangi91srivastava@gmail.com (S.S.); widmanns1020@gmail.com (S.W.); razegun@live.com (C.H.); donovan998@gmail.com (D.N.); alexisnguyen0825@gmail.com (A.N.); asitha078490@gmail.com (A.P.); jgustafs@Central.UH.EDU (J.-Å.G.)

**Keywords:** pancreatic cancer, liver X receptor, ligand, glutamine metabolism, oxidative stress

## Abstract

Pancreatic ductal adenocarcinoma (PDAC) is the predominant form of pancreatic cancer with a high mortality rate due to the lack of early detection and effective treatment options for advanced diseases. Metabolic reprogramming, a common hallmark of malignant transformation in pancreatic cancer, is critical for the growth and survival of cancer cells and a potential target mechanism for the treatment of pancreatic cancer. PDAC cells have upregulated glutamine metabolism to meet their biosynthetic and oxidative demands. Liver X receptors (LXRs) are ligand-dependent transcription factors involved in maintaining metabolic homeostasis. LXRs regulate critical cancer-related processes and pathways, including cholesterol, glucose and lipid metabolism, and inflammatory and immune responses. Analysis of transcriptomic data from PDAC clinical samples reveals overexpression of LXRs and their target genes in tumors as compared to normal tissue controls. Targeting LXRs with the novel LXR inverse agonist and degrader GAC0001E5 inhibited PDAC cell proliferation. Using a metabolomics approach, we discovered that 1E5 inhibits glutamine anaplerosis and induces oxidative stress, which are detrimental to PDAC cells. These findings highlight a novel role for LXR in regulating cancer metabolism and the potential application of LXR modulators in targeting cancer metabolism in pancreatic cancer and other malignancies.

## 1. Introduction

Pancreatic ductal adenocarcinoma (PDAC) is the third leading cause of cancer-related deaths in the United States, with a 5-year survival rate of 9.3% [1]. PDAC has a poor prognosis due to its aggressive nature, late diagnosis, and lack of effective therapeutic options for advanced disease. Currently, only 20% of patients present with localized tumors that are resected through surgery. The standard-of-care chemotherapy gemcitabine is the first-line treatment in patients with resected pancreatic cancer. The combination treatment of fluorouracil, leucovorin, irinotecan, and oxaliplatin (FOLFIRINOX) has a longer overall survival than gemcitabine alone in patients with metastatic pancreatic cancer, but overall efficacy, toxicity, and development of resistance remain significant challenges [2,3].

Liver X receptors (LXRs), i.e., LXRα and LXRβ, are nuclear receptors that regulate transcription of genes involved in cholesterol, glucose, lipid metabolism, and inflammatory responses [4]. LXRs form heterodimers with retinoid X receptors (RXRs) and bind to specific DNA recognition sequences on the target genes. LXR activity can be modulated by endogenous ligands such as oxysterols and synthetic ligands that have been developed to target LXRs in metabolic diseases, including atherosclerosis [5]. LXRs have also emerged as druggable targets in cancer therapeutics, and LXR ligands have been shown to elicit antiproliferative effects in multiple cancers, including in our previously published work on pancreatic cancer [6,7]. Recently, we identified a novel LXR modulator, GAC0001E5 (1E5), with more potent antitumor activity against PDAC cells than the current LXR agonist [8]. Compound 1E5 functions as an LXR inverse agonist and as a degrader that decreases LXR protein levels over time. Interestingly, the LXR inverse agonist SR9243 has been shown to inhibit cancer growth by downregulating aerobic glycolysis and fatty acid synthesis and to also affect tumor immunity [9,10]. Targeting cancer metabolism is a promising therapeutic strategy, and metabolic reprogramming driven by oncogenes enables PDACs to survive in hypoxic and nutrient-deprived microenvironments [11]. For example, oncogenic KRAS, present in more than 95% of PDAC tumors, upregulates glutamine metabolism for supporting its biosynthetic needs and maintaining redox homeostasis [12,13]. Glutamine is indispensable for PDAC growth as it serves as a carbon donor for TCA cycle anaplerosis and nitrogen donor for nucleotide biosynthesis and amino acid generation [14]. Additionally, glutamine-derived glutamate is a substrate for glutathione biosynthesis to counter oxidative stress in tumors [15,16]. Therefore, targeting glutamine metabolism is an attractive strategy in pancreatic cancer.

Here we show that the expression of LXR and its target metabolic genes are upregulated in PDACs. Treatments with a novel LXR modulator, 1E5, inhibits PDAC cell proliferation and sensitizes cells to gemcitabine. We further demonstrate that 1E5 disrupts glutamine metabolism and induces oxidative stress in PDAC cells. These findings highlight the therapeutic potential of targeting cancer metabolism through the modulation of LXRs in pancreatic cancer.

## 2. Results

### 2.1. LXRβ Is Overexpressed in Pancreatic Cancer and LXR Ligand 1E5 Inhibits PDAC Cell Proliferation

**Figure 1:**
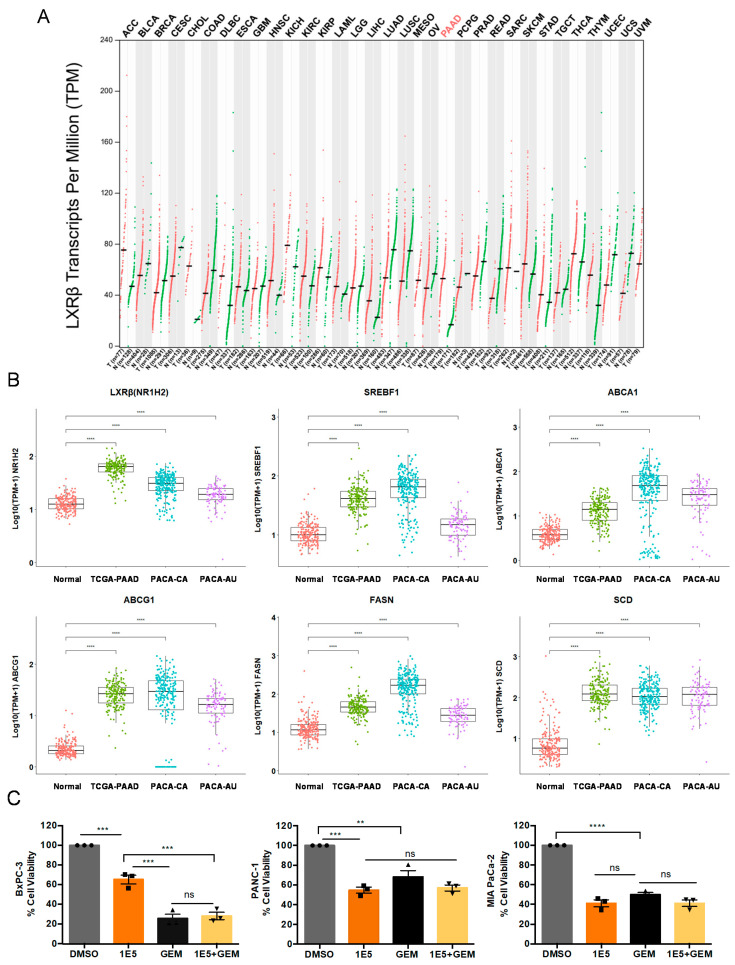
Pancreatic cancer cells overexpress *LXRβ* and are vulnerable to novel liver X receptor (LXR) modulators that function as inverse agonists and degraders. (**A**) Transcripts per million (TPM) of LXRβ was plotted for 33 different TCGA cohorts and their corresponding normal tissues. While LXRβ showed differential expression in a number of cancers, only the overexpression in pancreatic cancer cohort (PAAD) was statistically significant. (**B**) Log-normalized TPM expression of LXRβ and known target genes *SREBF1*, *ABCA1*, *ABCG1*, *FASN*, and *SCD* from three cancer cohorts, namely The Cancer Genome Atlas-Pancreatic Adenocarcinoma (TCGA-PAAD), Pancreatic Cancer-Canada (PACA-CA), and Pancreatic Cancer-Australia (PACA-AU), were compared to normal pancreatic tissues from The Genotype-Tissue Expression Project (GTEx) and all consistently showed overexpression in tumors as compared to normal tissues. (**C**) Treatment with novel LXR ligand 1E5 elicits antiproliferative response in pancreatic ductal adenocarcinoma (PDAC) cells. MTT assay of PDAC cells treated with DMSO, 1E5 (10 µM), gemcitabine (20 nM in BxPC-3, 40 nM in PANC-1 and MIA PaCa-2 cells), and 1E5 + Gem (5 µM + 10 nM gemcitabine in BxPC-3, 5 µM + 20 nM gemcitabine in PANC-1 and MIA PaCa-2 cells) for 72 h. Data presented as relative percentage to vehicle (DMSO)-treated cells. Data represent mean ± SEM, *n* = 3 independent biological replicates, ** *p* < 0.01, *** *p* < 0.001, **** *p* < 0.0001, not significant (ns) *p* > 0.05.

We have previously shown that LXRβ is expressed in PDAC cells [7]. To determine the potential clinical significance of LXRβ in human cancers, we examined its expression across 33 different cancer types using the Gene Expression Profiling Interactive Analysis (GEPIA) tool. Of the cancer types included in the analysis, only in the pancreatic cancer cohort (PAAD) were *LXRβ* transcript levels significantly elevated in the tumors as compared to normal pancreas (Figure 1A). To validate these results, we further investigated the transcriptomes of three pancreatic cancer cohorts, namely The Cancer Genome Atlas-Pancreatic Adenocarcinoma (TCGA-PAAD), Pancreatic Cancer-Canada (PACA-CA), and Pancreatic Cancer-Australia (PACA-AU), as well as normal pancreas tissues from The Genotype-Tissue Expression Project (GTEx) collection. We also compared the expression of *LXRβ* and known target genes *SREBF1*, *ABCA1*, *ABCG1*, *FASN*, and *SCD* in pancreatic cancer tissue to normal pancreas tissue and found that transcript levels of LXRβ and its target genes are elevated in the tumor tissues (Figure 1B).

Our recent study identified a novel LXR ligand, GAC0001E5 (1E5), that inhibits PDAC cell growth and survival in a dose-dependent manner [8]. Gemcitabine, a DNA synthesis inhibitor, is the standard of care for PDAC patients. However, the development of drug resistance limits its efficacy [3]. To determine the role of LXRβ in PDAC and whether combination treatment with 1E5 can enhance the sensitivity to gemcitabine, we measured the sensitivity of PDAC cells to 1E5 and gemcitabine alone and additively with half of their concentrations. Treatments with 1E5 significantly decreased proliferation of BxPC-3 (KRAS wt), PANC-1 (KRAS G12D mut), and MIA PaCa-2 (KRAS G12C mut) cells (see Figure 1C). Sensitivity to 1E5 appears to be cell line-dependent with PANC-1 and MIA PaCa-2 cells exhibiting greater sensitivity than BxPC-3 cells, which is consistent with our recent study [8]. We also observed variable sensitivity to gemcitabine where BxPC-3 cells were most sensitive and PANC-1 cells least sensitive. This is in line with a recent study showing that PANC-1 cells are resistant to gemcitabine due to higher nuclear factor erythroid 2-related factor 2 (NRF2) expression (17). Notably, combination treatments of 1E5 and gemcitabine using half of their individual concentrations elicited additive effects, comparable to individual treatments at full concentration, in all three cell lines (Figure 1C). This signifies the role of using LXR ligands to target pancreatic cancer proliferation in combination with existing chemotherapies.

Since LXRs regulate multiple metabolic pathways, we posit that the mechanism of action of 1E5 involves perturbation of tumor cell metabolism. To test this hypothesis, we conducted a metabolomic analysis using reverse-phase ultra-performance liquid chromatography (Metabolon) of two PDAC cell lines, BxPC-3 and PANC-1, treated with vehicle (DMSO), LXR agonist GW3965, or 1E5 for 48 h. Compounds were identified by comparison to Metabolon’s entries of purified standards or recurrent unknown entities. Using one-way ANOVA analysis (*p* ≤ 0.05), we identified metabolites differentially altered in BxPC-3 and PANC-1 cells upon GW3965 and 1E5 treatment. A total of 539 and 595 metabolites were significantly altered in BxPC-3 and PANC-1 cell lines, respectively (*p* ≤ 0.05). In BxPC-3 cells, with respect to DMSO, GW3965 treatment led to significant upregulation of 83 metabolites and downregulation of 367 metabolites whereas 1E5 treatment led to upregulation of 97 metabolites and downregulation of 142 metabolites. In PANC-1 cells, however, treatment with GW3965 led to upregulation of 216 metabolites and downregulation of 81, whereas 1E5 caused upregulation of 177 metabolites and downregulation of 187 (Table S1, Supplementary Materials).

Hierarchical clustering of samples based on their metabolite profiles showed group-dependent clustering of treatments in both BxPC-3 and PANC-1 cell lines. GW3965- and 1E5-treated BxPC-3 and PANC-1 cells showed clear separation of groups indicative of differential metabolic effects exerted by these ligands (Figure S1A,B). These results suggest that LXR agonist GW3965 and 1E5 have distinct metabolic profiles in wild-type KRAS and mutant KRAS cell lines. We also carried out RNAseq analysis to determine the transcriptomic changes in PANC-1 cells in response to 1E5 treatment. Gene set enrichment analysis (GSEA) revealed that downregulated pathways included cell cycle, fatty acid and lipid metabolism, oxidative phosphorylation, glycolysis, and mRNA splicing (Figure S2A). The upregulated pathways included unfolded protein response and peptide elongation pathway, which can be induced by the disruption of amino acid metabolism [14]. Disruption of these cancer-related pathways is in line with the antiproliferative effect of 1E5 treatment.

### 2.2. LXR Inverse Agonist and Degrader 1E5 Is a Potent Inhibitor of Glutamine Metabolism

**Figure 2:**
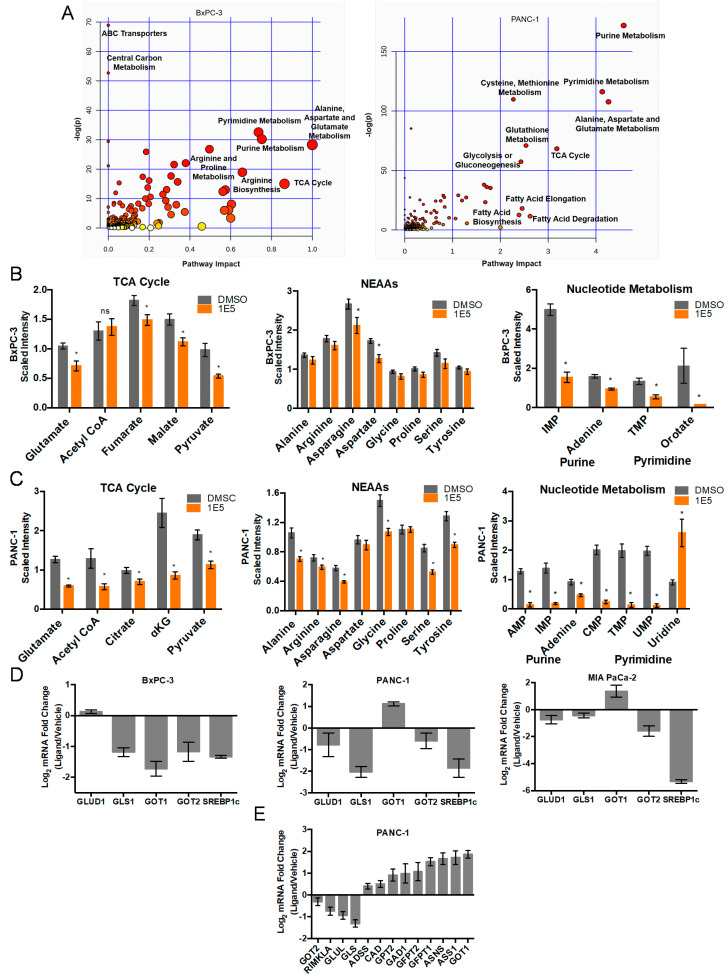
Treatments with 1E5 downregulate key metabolites and genes involved in glutamine metabolism in PDAC cells. (**A**) Overview of joint pathway analysis. The scatterplot represents the pathway impact value and *p*-value from joint pathway analysis of differentially expressed genes and metabolites from BxPC-3 and PANC-1 cells treated with 1E5 for 48 h. The size and color of each node is based on its pathway impact value and *p*-value, respectively. Pathways with statistical significance (*p* < 0.05) are shown in red. (**B**,**C**) TCA cycle, NEAAs, and nucleotide metabolism metabolite levels quantified by UPLC-MS/MS after 48 h treatment with DMSO and 1E5 (10 µM) in BxPC-3 (*n* = 5) and PANC-1 (*n* = 6) cells, respectively. Data were scaled to median intensity and normalized to cell number. Statistical significance determined by *t*-test, where * *p* < 0.05, not significant (ns) *p* > 0.05. (**D**) Gene expression analysis following 48 h treatment with 1E5 (10 µM). Data presented as mRNA fold change normalized to DMSO, data represent mean ± SEM, *n* = 3 independent biological replicates. (**E**) RNAseq differential gene expression analysis of glutamine metabolism genes in PANC-1 cells. Data presented as mRNA fold change normalized to DMSO, data represent mean ± SEM, *n* = 3 independent biological replicates.

To more comprehensively determine metabolic effects and mechanisms of 1E5 action, we carried out an integrated pathway analysis of metabolomics and RNAseq data using MetaboAnalyst 4.0 software [17]. The metabolic impact and pathway analysis demonstrated that pathways involved in ABC transporters, purine and pyrimidine metabolism, glutamate–aspartate metabolism, proline and arginine, glutathione metabolism, and TCA cycle were most affected in BxPC-3 cells. In PANC-1 cells, purine and pyrimidine metabolism, glutamate–aspartate metabolism, cysteine and glutathione metabolism, TCA cycle, and glucose metabolism were most affected upon 1E5 treatment (Figure 2A; Tables S2 and S3). PDACs rewire their metabolism to upregulate glutamine consumption to fuel TCA cycle anaplerosis as well as nucleotide and amino acid biosynthesis that are required for cancer cell proliferation [18]. The first step of glutamine metabolism is carried out by glutaminase (*GLS*), which converts glutamine to glutamate. Inhibiting GLS to target glutamine metabolism has been shown to decrease cell proliferation in multiple cancers [19]. Given the indispensable role of glutamine metabolism in PDAC biology, we focused our follow-up studies on the impact of 1E5 treatments on glutamine metabolism. Glutamate levels were significantly downregulated by 1E5 in both BxPC-3 and PANC-1 cells. TCA cycle intermediates, non-essential amino acids (NEAAs), and purine and pyrimidine metabolites were similarly downregulated in PDAC cells (Figure 2B,C). Notably, 1E5 had a more pronounced effect of downregulating glutamine metabolism in PANC-1 cells than in BxPC-3 cells. This observation is in line with the role of mutant KRAS in upregulating glutamine metabolism [20]. Next, we examined the expression levels of key glutamine metabolism genes using qPCR in three PDAC cell lines. Consistent with the observed reduced glutamate levels, treatments with 1E5 significantly downregulated GLS in all three cell lines. Glutamate oxaloacetate transaminase 2 (*GOT2*) transcript levels were also downregulated by 1E5 in the PDAC cell lines. In BxPC-3 cells, 1E5 marginally increased glutamate dehydrogenase 1 (GLUD1) and decreased glutamate oxaloacetate transaminase 1 (*GOT1*) expression. In contrast, in PANC-1 and MIA PaCa-2 cells, 1E5 decreased *GLUD1* and increased *GOT1* expression. Reduction in *SREBP1c* levels in PDAC cells further confirmed that 1E5 is an LXR inverse agonist (Figure 2D). Downregulation of *GLUD1* and *GOT2* in PANC-1 cells is consistent with the decrease in TCA cycle intermediates and NEAAs. We carried out RNAseq analysis in PANC-1 cells treated with 1E5. The data showed that 1E5 significantly altered the transcript profile of key glutamine metabolism genes, confirming the qPCR data (Figure 2E). Additionally, RNAseq data indicated that 1E5 treatment downregulated glutamine synthetase (*GLUL*) expression, a commonly upregulated gene in PDACs [12]. RNAseq data further revealed that 1E5 upregulated glutamine-independent glutamate-producing enzymes such as asparagine synthetase (*ASNS*), suggestive of the compensatory effects following 1E5 treatment [16,21]. Collectively, metabolomics and RNAseq data strongly suggest that 1E5 disrupts glutamine metabolism in PDAC cells.

**Figure 3:**
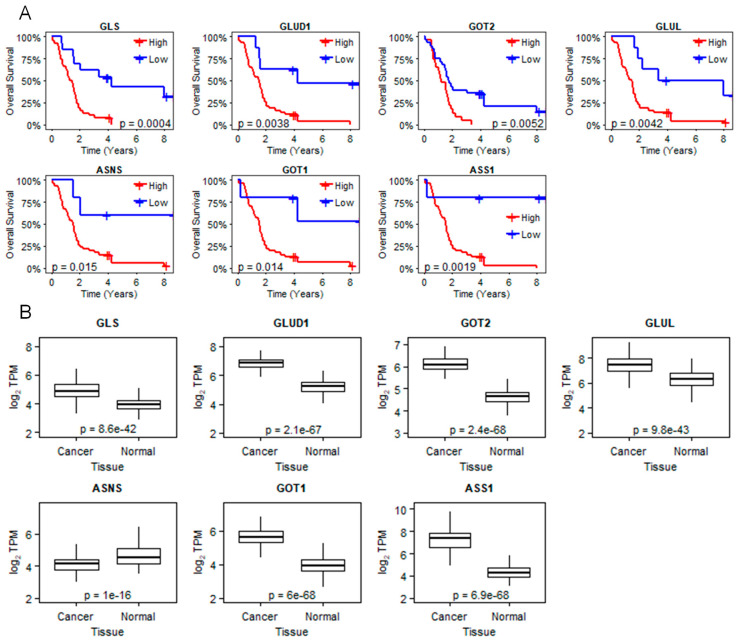
Key glutamine metabolism genes are differentially expressed in PDAC. (**A**) Kaplan–Meier survival plots of patients from the Kirby et al. dataset (*n* = 51). Patients are grouped by high or low expression of glutamine metabolism genes in PDAC patients and their overall survival probabilities over time are displayed. *p*-values from the log-rank tests for survival differences between high and low expression patient groups are reported. (**B**) Comparative gene analysis of pancreatic cancer samples (*n* = 270) and normal tissue. Log2 transformed TPM values of key genes involved in glutamine metabolism. Results were obtained from the GTEx portal.

To determine the potential clinical relevance of the glutamine metabolism genes regulated by the novel ligand, we analyzed gene expression profiles and patient survival data from the study published by Kirby and colleagues (*n* = 51) [22]. Higher expression of *GLS*, *GLUD1*, *GOT2*, *GLUL*, and *ASNS* genes correlates with poor survival of PDAC patients (Figure 3A). We also compared the expression levels of glutamine metabolism genes altered by 1E5 between cancer and normal tissue. Genes downregulated by 1E5 are overexpressed in cancer tissue, whereas *ASNS*, which is upregulated by 1E5, is downregulated in cancer tissue suggesting that genes altered by 1E5 are differentially expressed in tumors and highlight their potential roles in PDAC biology and disease progression (Figure 3B).

### 2.3. Combination Treatment of 1E5 with GLS Inhibitor BPTES Synergistically Decreases Glutaminolysis and Growth of PDAC Cells

**Figure 4:**
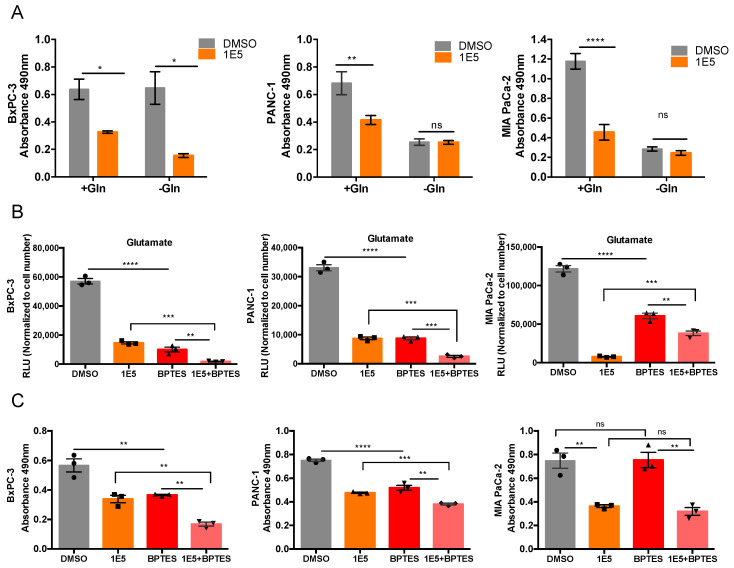
Combination treatment of 1E5 with a GLS inhibitor, BPTES, synergistically decreases intracellular glutamate levels and cell viability of PDAC cells. (**A**) MTT assay of PDAC cells cultured in media with or without 4 mM glutamine for 72 h. Data represent ± SEM and statistical significance determined by two-way ANOVA where * *p* < 0.01, ** *p* < 0.001, **** *p* < 0.0001, not significant (ns) *p* > 0.05. (**B**) Intracellular glutamate levels upon 48 h treatment with DMSO, 1E5 (10 µM), BPTES (10 µM), 1E5 (5 µM) + BPTES (5 µM) in BxPC-3, PANC-1, and MIA PaCa-2 cells. (**C**) Cell viability test in BxPC-3, PANC-1, and MIA PaCa-2 cells, respectively, *n* = 3 independent biological replicates. Data represent ± SEM and statistical significance determined by one-way ANOVA, where ** *p* < 0.01, *** *p* < 0.001, **** *p* < 0.0001, not significant (ns) *p* > 0.05.

Since PDAC cells are highly dependent on glutamine for their biosynthetic needs, and 1E5 downregulates multiple metabolites and genes involved in glutamine metabolism, we hypothesized that 1E5 treatments might mimic glutamine starvation conditions for PDAC cells in culture. To test this, we treated PDAC cells with 1E5 in glutamine-containing and glutamine-free media and assessed their growth using MTT assay. Glutamine starvation inhibited PANC-1 and MIA PaCa-2 proliferation but did not affect BxPC-3 cell growth. Treatments with 1E5 combined with glutamine starvation had no additive effect on cell proliferation of PANC-1 and MIA PaCa-2 cells, whereas it significantly inhibited BxPC-3 growth (Figure 4A). BxPC-3 cells utilize glucose as a preferential carbon source, and this may explain the antiproliferative effect of 1E5 treatment in glutamine-free media [23,24]. Inhibiting GLS alone has limited efficacy in PDAC tumors [16]. Therefore, we examined the combinatorial effects of 1E5 with GLS inhibitor BPTES, using half of their individual concentrations, on glutamate levels and PDAC cell proliferation. In BxPC-3 and PANC-1 cells, 1E5 alone significantly decreased the intracellular glutamate levels, comparable to BPTES treatment. Combination treatment showed a synergistic effect on the reduction of glutamate levels (Figure 4B). In MIA PaCa-2 cells, 1E5 had a more significant reduction than BPTES alone (Figure 4B). Next, we determined the effects of combination treatments on cell proliferation. Either compound alone significantly reduced proliferation of BxPC-3 and PANC-1 cells, and their combination showed a synergistic effect. Similar to observed glutamate levels in MIA PaCa-2 cells, 1E5 significantly reduced cell growth, but combination treatments did not elicit a synergistic response (Figure 4C). These results indicate that 1E5 potentiates the efficacy of GLS inhibition in PDAC cells.

### 2.4. 1E5 Treatment Induces Oxidative Stress in PDAC Cells

**Figure 5:**
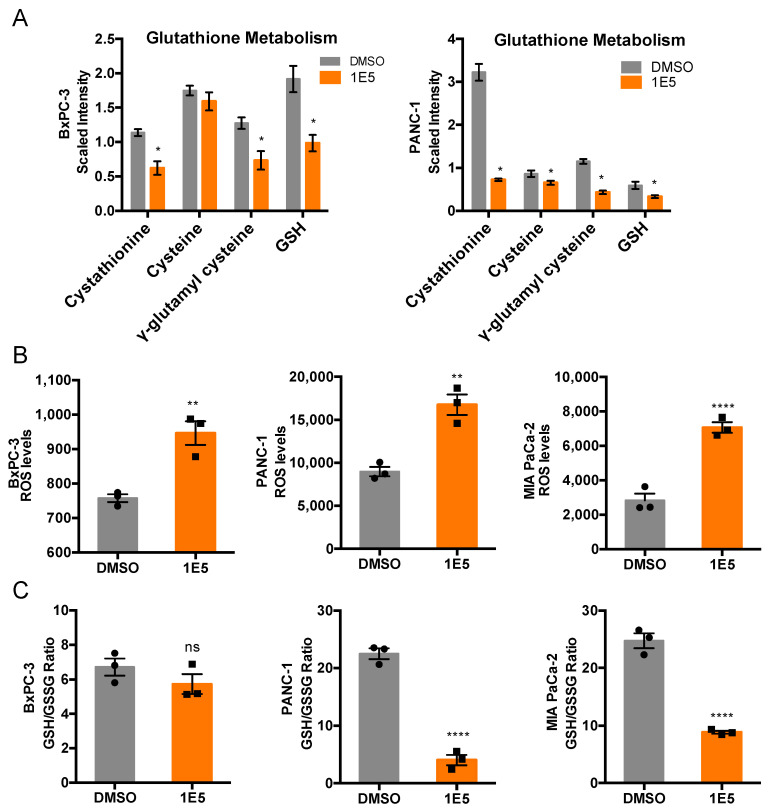
1E5 treatment induces oxidative stress response in BxPC-3 and PANC-1 cells. (**A**) Overview of the transsulfuration pathway and glutathione biosynthesis in cells. (**B**,**C**) Glutathione metabolism metabolite levels quantified by mass spectrometry in BxPC-3 (*n* = 5) and PANC-1 (*n* = 6) cells following 48 h treatment with DMSO and 1E5. Statistical significance determined by *t*-test, where * *p* < 0.05. (**B**) H_2_O_2_ luminescence normalized by cell number following 48 h treatment with DMSO and 1E5 in BxPC-3, PANC-1, and MIA PaCa-2 cells. (**C**) Ratio of reduced to oxidized glutathione (GSH/GSSG) following treatment with DMSO and 1E5 in HBSS buffer for 48 h. Data representative of mean ± SEM, *n* = 3 independent biological replicates. Statistical significance determined by one-way ANOVA, where * *p* < 0.01, ** *p* < 0.001, **** *p* < 0.0001, ns *p* > 0.05.

Glutathione (GSH), a tripeptide composed of glutamate, cysteine, and glycine, plays a vital role in detoxification of electrophilic compounds [25]. We reasoned that a decrease in glutamate levels in response to 1E5 treatment could downregulate GSH levels and induce oxidative stress-induced cell death in PDAC cells. Indeed, 1E5 reduced the intracellular levels of GSH and its precursor γ-glutamyl cysteine in both BxPC-3 and PANC-1 cells. We also noted cysteine levels that were synthesized through transsulfuration (TSS) of methionine via cystathionine [25]. TSS pathway has been shown to have a pro-tumorigenic function by maintaining cellular GSH levels [26]. Importantly, 1E5 treatment inhibited TSS pathway by significantly decreasing cystathionine levels in BxPC-3 and PANC-1 cells and cysteine levels in PANC-1 cells (Figure 5A). We then assessed oxidative stress by measuring reactive oxygen species (ROS) and GSH/GSSG (reduced/oxidized glutathione) levels in control and 1E5-treated cells. As expected, BxPC-3, PANC-1, and MIA PaCa-2 cells showed significant upregulation of ROS levels (Figure 5B). Interestingly, BxPC-3 cells showed no significant change in intracellular GSH/GSSG levels, but in PANC-1 and MIA PaCa-2 cells, GSH/GSSG levels were significantly reduced (Figure 5C). These results indicate that 1E5 induces oxidative stress by increasing ROS levels and decreasing GSH levels in PDAC cells.

## 3. Discussion

PDAC is an aggressive disease where cancer cells have reprogrammed their metabolism in order to fulfill their energetic and biosynthetic demands [13]. Glutamine “addiction” is one of the most prominent metabolic changes in PDAC cells, making it a potential target mechanism [11]. LXRs, key regulators of metabolism genes, have emerged as promising targets in cancer therapeutics. In this study, we showed that LXRβ and its target genes are overexpressed in pancreatic cancer and we applied an integrative -omics approach to characterize the mechanisms of action of a novel LXR modulator, 1E5, that we recently identified in a screen of putative LXR ligands for inhibitory activity in PDAC cells [8]. We showed that 1E5 inhibits glutamine metabolism, induces oxidative stress in PDAC cells, and provides a novel approach for disrupting glutamine utilization in PDAC (summarized in Figure S2B). The combinatorial strategy of inhibiting glutamine metabolism along with induction of oxidative stress has shown antitumor activity in PDACs [16,27]. Therefore, induction of oxidative stress and inhibition of glutamine metabolism upon 1E5 treatment are important findings that support the current efforts to target PDAC metabolism as a therapeutic approach.

Glutamine is a versatile amino acid where its metabolites are involved in the TCA cycle, amino acid synthesis, and nucleotide biosynthesis [14]. Here, we showed that 1E5 inhibits glutamine utilization in PDAC cells by downregulating glutamine metabolism pathways, including TCA cycle and synthetic pathways for NEAAs and nucleotides. These data indicate that targeting LXR disrupts glutamine metabolism in PDAC cells. Our observation is further supported by reports showing the effect of an LXR agonist, LXR623, on partially increased glutamine metabolism in glioblastoma cells and identifying GLS as an LXR target in macrophages [28,29]. Targeting glutamine metabolism using a GLS1 inhibitor, CB839, has shown limited efficacy in pancreatic cancer mouse models. One reason for the limited efficacy of this approach is that GLS1 is a proximal enzyme in the glutamine metabolism pathway, and inhibiting it alone upregulates compensatory pathways [16]. Consistent with this finding, our study demonstrates the synergistic antiproliferative effect of combination treatment of 1E5 and GLS1 inhibitor BPTES, highlighting the importance of targeting multiple glutamine utilization nodes to overcome the limited efficacy of GLS1 inhibition alone in pancreatic cancer. Furthermore, a recent study has shown that disrupting glutamine metabolism enhances PDAC cells’ sensitivity to chemotherapy gemcitabine [30]. In agreement, the combination treatment of 1E5 and gemcitabine using half of their concentrations additively decreased PDAC cell growth. Importantly, combining chemotherapy with a targeted approach such as LXR ligand 1E5 can help reduce the likelihood of chemoresistance. Further studies will be required to validate the combinatorial effect of 1E5 with gemcitabine and BPTES in other preclinical PDAC models and examine possible metabolic adaptations in response to 1E5 treatment.

In addition to its biosynthetic roles, glutamine-derived glutamate is required for GSH synthesis. GSH is the most abundant antioxidant in cells, which scavenges intracellular free radicals and detoxifies reactive compounds [25]. GSH is important in carcinogenesis and the development of drug resistance [26]. Cancer cells maintain redox homeostasis by upregulating glutamine metabolism to generate glutamate and TSS pathway for cysteine synthesis and for GSH synthesis [13,31]. Elevating ROS levels has been shown to have an antiproliferative effect in PDAC tumors and increased sensitivity to gemcitabine [32]. A recent study found that LXR agonist T0901317 protects Schwann cells from ROS-induced damage by upregulating the antioxidant program [33]. That study supports our observation that targeting LXR using the inverse agonist 1E5 induced ROS-mediated oxidative stress. Moreover, the role of LXRs in regulating transsulfuration is poorly understood, and LXR ligand 1E5 can be a valuable tool for studying their role in regulating the TSS pathway. Our observation that 1E5 has a more potent effect in KRAS mutant cells than KRAS wild-type cells further supports the current efforts in targeting mutant KRAS-induced pathways in pancreatic cancer [20]. Identifying novel therapeutic targets and strategies to overcome resistance to chemotherapies such as gemcitabine is a significant challenge in PDAC. This study specifically shows that targeting PDAC metabolism using 1E5 disrupts cell growth and reiterates the rationale for targeting LXR activity to exploit the metabolic dependency of pancreatic cancer cells and potentially in other malignancies as well.

## 4. Materials and Methods

### 4.1. Cell Lines and Culture

BxPC-3 cells were cultured in RPMI 1640 (Gibco, Thermo Fischer Scientific, Waltham, MA, USA 11875085). MIA PaCa-2 and PANC-1 cells were cultured in DMEM (Gibco, 12430047) containing high glucose and HEPES. All media were supplemented with 10% FBS (Gibco, 26140079). All cell lines were cultured in a humidified atmosphere of 5% CO_2_ at 37 °C. All cell lines were free from mycoplasma contamination and authenticated using short tandem repeat (STR) profiling (ATCC 135-XV). DMSO was purchased from VWR, Radnor, PA, USA (97063-136). GW3965 was purchased from Tocris Bioscience, Minneapolis, MN, USA (2474), 1E5 was synthesized and obtained from OTAVA Chemicals (Concord, ON, Canada) and BPTES was obtained from Cayman Chemicals, Ann Arbor, MI, USA (19284).

### 4.2. Cell Proliferation Assay

Cells were plated in a 96-well plate at densities of BxPC-3 (2500 cells/well), PANC-1 (3000 cells/well), and MIA PaCa-2 (3000 cells/well). The cells were treated with ligands at indicated concentrations for 72 h in a media containing 4 mM glutamine. The media was not replaced within 72 h. For glutamine starvation assay, the cells were treated in glutamine-free media, DMEM (Thermo Fischer Thermo Fischer Scientific, Waltham, MA, USA, 11960051) and RPMI (Thermo Fischer Scientific, Waltham, MA, USA Thermo Fischer, 21870076) supplemented with 10% dialyzed FBS. Post 72 h, cell growth was measured using MTT assay (3-(4,5-dimethylthiazol-2-yl)-5-(3-carboxymethoxyphenyl)-2-(4-sulfophenyl)-2H-tetrazolium, Promega, Madison, WI, USA G3581).

### 4.3. Real-Time Quantitative PCR

Cells were plated in 6-well plates at densities of BxPC-3 and MIA PaCa-2 (250,000 cells/well), and PANC-1 (200,000 cells/well). After 24 h, cells were treated with vehicle (DMSO) and 1E5 (10 µM) for 48 h in a complete media. Total RNA was extracted using an RNeasy Kit (Qiagen Inc., Germantown, MD, USA) following the manufacturer’s directions. Then, 1 µg RNA was used for reverse transcription using iScript reverse transcriptase (Bio-Rad, Hercules, CA, USA 1708890) following the manufacturer’s directions. qPCR was performed with Power Up SYBR Green Master Mix (Thermo Fischer Scientific, A25742) on an Applied Biosystem 7500 (Thermo Fischer Scientific, Waltham, MA, USA) Fast PCR Machine. RPLPO gene was used as an internal control. A minimum of three biological replicates were conducted for each cell line. Relative fold change (to vehicle) was determined using the ΔΔCt method.

The primer sequences for the glutamine metabolism genes are as follows:

- 36B4-Forward: 5′-GTGTTCGACAATGGCAGCAT-3′
- 36B4-Reverse: 5′-GACACCCTCCAGGAAGCGA-3′
- GLUD1-Forward: 5′-AGGAATGACACCAGGGTTTG-3′
- GLUD1-Reverse: 5′-GAGGGTTGGAATACATGGGAC-3′
- GLS1-Forward: 5′-TTCCAGAAGGCACAGACATG-3′
- GLS1-Reverse: 5′-GGCTCAGTACTCTTTCACCAG-3′
- GOT1-Forward: 5′-CAACTGGGATTGACCCAACT-3′
- GOT1-Reverse: 5′-GGAACAGAAACCGGTGCTT-3′
- GOT2-Forward: 5′-GTTTGCCTCTGCCAATCATATG-3′
- GOT2-Reverse: 5′-GAGGGTTGGAATACATGGGAC-3′
- SREBP-1c-Forward: 5′-GGAGGGGTAGGGCCAACGGCCT-3′
- SREBP-1c-Reverse: 5′-CATGTCTTCGAAAGTGCAATCC-3′

### 4.4. Metabolomics

Metabolomics analysis was performed by Metabolon, Inc. (Durham, NC, USA). BxPC-3 and PANC-1 cells were grown in 10 cm plates and treated with DMSO, GW3965 (10 µM), and 1E5 (10 µM) for 48 h in a medium containing 4 mM glutamine and 25 g/L glucose supplemented with 10% FBS, *n* = 6. Briefly, samples were homogenized and subjected to methanol extraction and then split into aliquots for analysis by ultrahigh-performance liquid chromatography/mass spectrometry (UHPLC/MS) in the positive, negative, or polar ion mode. Metabolites were identified by automated comparison of ion features to a reference library of chemical standards followed by visual inspection for quality control as previously described [34,35,36]. For statistical analysis and data display, any missing values were assumed to be below the limits of detection; these values were imputed with the compound minimum. Data were normalized to cell number to account for differences in metabolite levels because of the differences in cell number due to treatments.

Statistical tests were performed in ArayStudio (Omicsoft) (http://omicsoft.com/software/ArrayStudioLauncher/publish.htm) or R to compare data between experimental groups; *p* < 0.05 was considered significant. An estimate of the false discovery rate (FDR) (Q-value) was also calculated to consider the multiple comparisons that normally occur in metabolomic-based studies, with Q < 0.05 used as an indication of high confidence in a result.

### 4.5. Intracellular Glutamate Assay

Intracellular glutamate levels were detected using a Promega Glutamate-Glo Assay Kit, Madison, WI, USA(J7021). Briefly, PANC-1, BxPC-3, and MIA PaCa-2 cells (10,000 cells/well) were plated in a 96-well plate in a complete medium. The following day, cells were treated with vehicle, 1E5, BPTES, and 1E5 + BPTES for 48 h in a media containing 5 mM glucose and 2 mM glutamine at indicated concentrations. Post 48 h, cells were washed twice with PBS and 30 µL PBS was added to each well. The cells were lysed by adding 15 µL of 0.3N HCl solution. The plate was shaken for 5 min at 100 rpm and lysing reagent was inactivated by adding 15 µL of 450mM Tris (pH 8.0) to each well. Then, a 12.5 µL portion of the sample was added to a 384-well plate followed by 12.5 µL of glutaminase in glutaminase buffer. The plate was shaken for 1 min and incubated for 40 min at room temperature (RT). After the incubation, a 50 µL portion of glutamate detection reagent was added and the plate was incubated at RT for 60 min. The luminescence was recorded using a Victor X4 plate reader (PerkinElmer, Waltham, MA, USA). All values were normalized to cell number identified by MTT reagent.

### 4.6. ROS Measurement

ROS levels were detected using an ROS-Glo H_2_O_2_ Assay (Promega, Madison, WI, USA G8820). Briefly, PANC-1, BxPC-3, and MIA PaCa-2 cells (10,000 cells/well) were plated in a 96-well plate in a complete medium. The following day, cells were treated with DMSO and 1E5 (10μM). In the last 6 h of the 48 h treatment, a 20 µL portion of 125 µM H_2_O_2_ substrate solution was added to each well. The plate was incubated at RT for 20 min and luminescence was recorded using a Victor X4 plate reader (PerkinElmer, Waltham, MA). All values were normalized to cell number.

### 4.7. GSH/GSSG Assay

GSH/GSSG levels were measured using a GSH/GSSG-Glo Assay (Promega, Madison, WI, USA V6611). Briefly, PANC-1, BxPC-3, and MIA PaCa-2 cells (5000 cells/well) were plated in a 96-well plate in a complete medium. The following day, cells were treated with DMSO and 1E5 (10 µM) in HBS buffer for 48 h. Post treatment, the wells were washed with PBS followed by addition of 50 µL total/oxidized glutathione lysis reagent. The plate was shaken at RT for 5 min and a 50 µL portion of luciferin generation reagent was added to each well. The plate was incubated at RT for 30 min followed by addition of 100 µL luciferin detection reagent. After 15 min of incubation, luminescence was recorded using a Victor X4 plate reader (PerkinElmer, Waltham, MA, USA). All values were normalized to cell number.

### 4.8. RNAseq Library Preparation and Sequencing

RNA samples underwent quality control assessment using the RNA tape on Tapestation 4200 (Agilent Technologies Inc., Santa Clara, CA, USA) and were quantified with a Qubit Fluorometer (Thermo Fisher). The RNA libraries were prepared and sequenced at the University of Houston Seq-N-Edit Core per standard protocols. RNA libraries were prepared with a QIAseq Stranded Total RNA Library Kit (Qiagen Inc., Germantown, MD, USA) using 500 ng input RNA. mRNA was enriched with Oligo-dT probes attached to Pure mRNA beads (Qiagen Inc., Germantown, MD, USA). RNA was fragmented, reverse transcribed into cDNA, and ligated with Illumina sequencing adaptors. The size selection for libraries was performed using SPRIselect beads (Beckman Coulter, Brea, CA, USA) and purity of the libraries was analyzed using the DNA 1000 tape Tapestation 4200 (Agilent Technologies Inc., Santa Clara, CA, USA). The indexed libraries were pooled and 2 × 76 bp paired-end reads were sequenced using NextSeq 500 (Illumina Technologies, Calistoga, CA, USA).

### 4.9. RNAseq Analysis

Paired-end FASTQ files were adapter- and quality-trimmed with Trim Galore v.0.4.4 and aligned to indexes built using Homo_sapiens.GRCh38.dna.primary_assembly with HISAT2 v.2.0.2 [37]. SAM files were sorted and converted to BAM format using SAM tools v.1.3. Transcripts were assembled and quantified with Homo_sapiens.GRCh38.99.gtf using StringTie v.1.3.1c. Transcript matrices were imported into RStudio v.1.2.1355 and differential expression was performed with DESeq2 v.1.22.2. Pre-ranked GSEA v.4.0.3 was performed on FPKM-filtered (Fragments per kilobase) (mean ≥ 1 across all samples) genes using the Wald test statistic from differential expression analysis and the c2.cp.v7.0. symbols gene set. EnrichmentMap [38] v.3.2.1 plugin for Cytoscape [39] v.3.7.2 was used with FDR < 0.05 and an overlap coefficient of 0.5.

### 4.10. Survival Analysis

The Cancer Genome Atlas (TCGA) RNA-seq HTSeq raw gene-level counts and clinical data (*n* = 176) for the pancreatic adenocarcinoma project (PAAD) were obtained with the package TCGA biolinks (v.3.11), and RNA-seq raw gene-level counts and clinical data (*n* = 51) from Kirby et al. 2016 were obtained from the Gene Expression Omnibus (GEO) using accession number GSE79668. Raw counts were converted to transcripts per million (TPM) using gene lengths derived from the feature counts function with Homo_sapiens.GRCh38.99.gtf.gz in Rsubread7. Kaplan–Meier survival curves were created using the survminer8 package [40,41] (v.0.4.7). Cutoff points for stratification were made using the surv_cutpoint function in survminer, which uses the maximally selected rank statistic to dichotomize TPM into high and low categories with the maxstat9 package. *p*-values from the log-rank test are reported.

For comparative gene expression analyses, The Gene Expression Profiling Interactive Analysis (GEPIA) web server (http://gepia.cancer-pku.cn/) was used to look at expression of *LXRβ* (NR1H2) transcripts across 33 cohorts and their corresponding normal tissues. Expression of *LXRβ* and its target genes across three pancreatic cancer cohorts, namely TCGA-PAAD (The Cancer Genome Atlas-Pancreatic Adenocarcinoma), PACA-CA (Pancreatic Cancer-Canada), and PACA-AU (Pancreatic Cancer-Australia), was compared to normal pancreas tissue from GTEx (The Genotype-Tissue Expression Project). TCGAbiolinks (2.12.6) was used to import TCGA-PAAD and GTEx raw counts in R (3.6.1), and the raw counts for PACA-CA and PACA-AU were downloaded from the International Cancer Genome Consortium (ICGC) data portal (https://dcc.icgc.org). The data were TPM normalized using gene lengths from human genome 38 (hg38) from Rsubread (1.34.7). The ggpubr (0.2.5) package in R was used for conducting Welch’s two-sample unpaired *t*-test between groups.

### 4.11. Statistics

All sample size (*n*) values used for statistical analyses are provided in the relevant figures and supplementary figures. For comparison of two groups, an unpaired two-tailed Student’s *t*-test or ANOVA was performed. Data are presented as the mean ± SEM. Statistical significance was set at *p* < 0.05. Statistical analyses were performed using GraphPad Prism 6.0.0 for MacOS, San Diego, CA, USA.

## Supplementary Materials

The following are available online at https://www.mdpi.com/1422-0067/21/24/9622/s1.

## Abbreviations

PDAC	Pancreatic ductal adenocarcinoma
LXR	Liver X receptor
RXR	Retinoid X receptor
GEM	Gemcitabine
GAC0001E5	1E5
GEPIA	Gene Expression Profiling Interactive Analysis
TCGA-PAAD	The Cancer Genome Atlas-Pancreatic Adenocarcinoma
PACA-CA	Pancreatic Cancer-Canada
PACA-AU	Pancreatic Cancer-Australia
GSEA	Gene set enrichment analysis
GLS	Glutaminase
NEAAs	Non-essential amino acids
GSH	Glutathione
ROS	Reactive oxygen species
TSS	Transsulfuration
TPM	Transcripts per million

## References

[1] Siegel R.L., Miller K.D., Jemal A. (2020). Cancer statistics, 2020. CA Cancer J. Clin..

[2] Conroy T., Hammel P., Hebbar M., Ben Abdelghani M., Wei A.C., Raoul J.-L., Choné L., Francois E., Artru P., Biagi J.J. (2018). FOLFIRINOX or Gemcitabine as adjuvant therapy for pancreatic cancer. N. Engl. J. Med..

[3] Principe D.R., Narbutis M., Kumar S., Park A., Viswakarma N., Dorman M.J., Kamath S.D., Grippo P.J., Fishel M.L., Hwang R.F. (2020). Long-term gemcitabine treatment reshapes the pancreatic tumor microenvironment and sensitizes murine carcinoma to combination immunotherapy. Cancer Res..

[4] Jakobsson T., Treuter E., Gustafsson J.A., Steffensen K.R. (2012). Liver X receptor biology and pharmacology: New pathways, challenges and opportunities. Trends Pharm. Sci..

[5] Lin C.Y., Gustafsson J.A. (2015). Targeting liver X receptors in cancer therapeutics. Nat. Rev. Cancer.

[6] Lin C.Y., Vedin L.L., Steffensen K.R. (2016). The emerging roles of liver X receptors and their ligands in cancer. Expert Opin. Ther. Targets.

[7] Candelaria N.R., Addanki S., Zheng J., Nguyen-Vu T., Karaboga H., Dey P., Gabbi C., Vedin L.L., Liu K., Wu W. (2014). Antiproliferative effects and mechanisms of liver X receptor ligands in pancreatic ductal adenocarcinoma cells. PLoS ONE.

[8] Karaboga H., Huang W., Srivastava S., Widmann S., Addanki S., Gamage K.T., Mazhar Z., Ebalunode J.O., Briggs J.M., Gustafsson J.-Å. (2020). Screening of focused compound library targeting liver X receptors in pancreatic cancer identified ligands with inverse agonist and degrader activity. ACS Chem. Biol..

[9] Pencheva N., Buss C.G., Posada J., Merghoub T., Tavazoie S.F. (2014). Broad-spectrum therapeutic suppression of metastatic melanoma through nuclear hormone receptor activation. Cell.

[10] Carpenter K.J., Valfort A.C., Steinauer N., Chatterjee A., Abuirqeba S., Majidi S., Sengupta M., Di Paolo R.J., Shornick L.P., Zhang J. (2019). LXR-inverse agonism stimulates immune-mediated tumor destruction by enhancing CD8 T-cell activity in triple negative breast cancer. Sci. Rep..

[11] Halbrook C.J., Lyssiotis C.A. (2017). Employing Metabolism to Improve the Diagnosis and Treatment of Pancreatic Cancer. Cancer Cell.

[12] Bott A.J., Shen J., Tonelli C., Zhan L., Sivaram N., Jiang Y.P., Yu X., Bhatt V., Chiles E., Zhong H. (2019). Glutamine anabolism plays a critical role in pancreatic cancer by coupling carbon and nitrogen metabolism. Cell Rep..

[13] Son J., Lyssiotis C.A., Ying H., Wang X., Hua S., Ligorio M., Perera R.M., Ferrone C.R., Mullarky E., Shyh-Chang N. (2013). Glutamine supports pancreatic cancer growth through a KRAS-regulated metabolic pathway. Nature.

[14] Yang L., Venneti S., Nagrath D. (2017). Glutaminolysis: A hallmark of cancer metabolism. Annu. Rev. Biomed. Eng..

[15] Vučetić M., Cormerais Y., Parks S.K., Pouysségur J. (2017). The Central Role of Amino Acids in Cancer Redox Homeostasis: Vulnerability Points of the Cancer Redox Code. Front. Oncol..

[16] Biancur D.E., Paulo J.A., Malachowska B., Quiles Del Rey M., Sousa C.M., Wang X., Sohn A.S.W., Chu G.C., Gygi S.P., Harper J.W. (2017). Compensatory metabolic networks in pancreatic cancers upon perturbation of glutamine metabolism. Nat. Commun..

[17] Chong J., Wishart D.S., Xia J. (2019). Using MetaboAnalyst 4.0 for comprehensive and integrative metabolomics data analysis. Curr. Protoc. Bioinform..

[18] Biancur D.E., Kimmelman A.C. (2018). The plasticity of pancreatic cancer metabolism in tumor progression and therapeutic resistance. Biochim. Biophys. Acta Rev. Cancer.

[19] Choi Y.K., Park K.G. (2018). Targeting Glutamine Metabolism for Cancer Treatment. Biomol. Ther..

[20] Bryant K.L., Mancias J.D., Kimmelman A.C., Der C.J. (2014). KRAS: Feeding pancreatic cancer proliferation. Trends Biochem. Sci..

[21] Cheng C.T., Qi Y., Wang Y.C., Chi K.K., Chung Y., Ouyang C., Chen Y.R., Oh M.E., Sheng X., Tang Y. (2018). Arginine starvation kills tumor cells through aspartate exhaustion and mitochondrial dysfunction. Commun. Biol..

[22] Kirby M.K., Ramaker R.C., Gertz J., Davis N.S., Johnston B.E., Oliver P.G., Sexton K.C., Greeno E.W., Christein J.D., Heslin M.J. (2016). RNA sequencing of pancreatic adenocarcinoma tumors yields novel expression patterns associated with long-term survival and reveals a role for ANGPTL4. Mol. Oncol..

[23] Tataranni T., Agriesti F., Ruggieri V., Mazzoccoli C., Simeon V., Laurenzana I., Scrima R., Pazienza V., Capitanio N., Piccoli C. (2017). Rewiring carbohydrate catabolism differentially affects survival of pancreatic cancer cell lines with diverse metabolic profiles. Oncotarget.

[24] Roux C., Riganti C., Borgogno S.F., Curto R., Curcio C., Catanzaro V., Digilio G., Padovan S., Puccinelli M.P., Isabello M. (2017). Endogenous glutamine decrease is associated with pancreatic cancer progression. Oncotarget.

[25] Bansal A., Simon M.C. (2018). Glutathione metabolism in cancer progression and treatment resistance. J. Cell Biol..

[26] Kalinina E.V., Gavriliuk L.A. (2020). Glutathione synthesis in cancer cells. Biochemistry.

[27] Chakrabarti G., Moore Z.R., Luo X., Ilcheva M., Ali A., Padanad M., Zhou Y., Xie Y., Burma S., Scaglioni P.P. (2015). Targeting glutamine metabolism sensitizes pancreatic cancer to PARP-driven metabolic catastrophe induced by ß-lapachone. Cancer Metab..

[28] Nguyen T.T.T., Ishida C.T., Shang E., Shu C., Torrini C., Zhang Y., Bianchetti E., Sanchez-Quintero M.J., Kleiner G., Quinzii C.M. (2019). Activation of LXRβ inhibits tumor respiration and is synthetically lethal with Bcl-xL inhibition. EMBO Mol. Med..

[29] Pehkonen P., Welter-Stahl L., Diwo J., Ryynänen J., Wienecke-Baldacchino A., Heikkinen S., Treuter E., Steffensen K.R., Carlberg C. (2012). Genome-wide landscape of liver X receptor chromatin binding and gene regulation in human macrophages. BMC Genomics.

[30] Mukhopadhyay S., Goswami D., Adiseshaiah P.P., Burgan W., Yi M., Guerin T.M., Kozlov S.V., Nissley D.V., McCormick F. (2020). Undermining glutaminolysis bolsters chemotherapy while NRF2 promotes chemoresistance in KRAS-driven pancreatic cancers. Cancer Res..

[31] Zhu J., Berisa M., Schwörer S., Qin W., Cross J.R., Thompson C.B. (2019). Transsulfuration Activity Can Support Cell Growth upon Extracellular Cysteine Limitation. Cell Metab..

[32] Donadelli M., Dando I., Zaniboni T., Costanzo C., Dalla Pozza E., Scupoli M.T., Scarpa A., Zappavigna S., Marra M., Abbruzzese A. (2011). Gemcitabine/cannabinoid combination triggers autophagy in pancreatic cancer cells through a ROS-mediated mechanism. Cell Death Dis..

[33] Hichor M., Sundaram V.K., Eid S.A., Abdel-Rassoul R., Petit P.X., Borderie D., Bastin J., Eid A.A., Manuel M., Grenier J. (2018). Liver X Receptor exerts a protective effect against the oxidative stress in the peripheral nerve. Sci. Rep..

[34] Evans A.M., Bridgewater B., Liu Q., Mitchell M., Robinson R., Dai H., Stewart S., Dehaven C.D., Miller L. (2014). High resolution mass spectrometry improves data quantity and quality as compared to unit mass resolution mass spectrometry in highthroughput profiling metabolomics. Metab. Open Access..

[35] Evans A.M., DeHaven C.D., Barrett T., Mitchell M., Milgram E. (2009). Integrated, nontargeted ultrahigh performance liquid chromatography/electrospray ionization tandem mass spectrometry platform for the identification and relative quantification of the small-molecule complement of biological systems. Anal. Chem..

[36] Dehaven C.D., Evans A.M., Dai H., Lawton K.A. (2010). Organization of GC/MS and LC/MS metabolomics data into chemical libraries. J. Cheminform..

[37] Pertea M., Kim D., Pertea G.M., Leek J.T., Salzberg S.L. (2016). Transcript-level expression analysis of RNA-seq experiments with HISAT, StringTie and Ballgown. Nat. Protoc..

[38] Merico D., Isserlin R., Stueker O., Emili A., Bader G.D. (2010). Enrichment map: A network-based method for gene-set enrichment visualization and interpretation. PLoS ONE.

[39] Shannon P., Markiel A., Ozier O., Baliga N.S., Wang J.T., Ramage D., Amin N., Schwikowski B., Ideker T. (2003). Cytoscape: A software environment for integrated models of biomolecular interaction networks. Genome Res..

[40] Li B., Ruotti V., Stewart R.M., Thomson J.A., Dewey C.N. (2010). RNA-Seq gene expression estimation with read mapping uncertainty. Bioinformatics.

[41] Liao Y., Smyth G.K., Shi W. (2019). The R package Rsubread is easier, faster, cheaper and better for alignment and quantification of RNA sequencing reads. Nucleic Acids Res..

