# Supplementary material for: Novel Liver X Receptor Ligand GAC0001E5 Disrupts Glutamine Metabolism and Induces Oxidative Stress in Pancreatic Cancer Cells
Source: Int J Mol Sci. 2020 Dec 17;21(24):9622. doi: 10.3390/ijms21249622 (PMC7767092; doi:10.3390/ijms21249622)
Supplement: Supplementary file 1 [file ijms-21-09622-s001.pdf]

Supplementary Figure Legends

Supplementary Figure S1: PDAC cells overexpress LXRβ and treatment with LXR agonist GW3965 and novel ligand 1E5 showed clear treatment dependent clustering of samples. **A, B** Hierarchical Component Analysis (HCA) plots of metabolic profiles of BxPC-3 and PANC-1 cells, (n=6).

Supplementary Figure S2: 1E5 treatment differential gene expression analysis identifies upregulated and downregulated pathways in PDAC cells. **A** Differential gene expression results from RNA-seq were filtered for FPKM > 1 in either treatment group. Genes were then ranked by the test statistic (Wald) from differential expression analysis. GSEA (version 4.0.3) was performed using the c2.cp.v7.0. symbols gene sets. GSEA results were plotted using Enrichment Map (version 3.2.1) with an FDR cutoff of 0.05 and overlap coefficient of 0.5. Red denotes positive enrichment while blue denotes negative enrichment. Darker border color indicates lower FDR. **B** Summary of LXR's novel ligand 1E5's mechanism of action in PDAC cells. 1E5 inhibits glutamine utilization by downregulating glutaminolysis and subsequent anaplerosis of glutamate into TCA cycle, NEAA synthesis and nucleotide biosynthesis. Additionally, by down-regulating glutathione levels 1E5 treatment induces oxidative stress in PDAC cells leading to inhibition of PDAC growth. Created with BioRender.com.

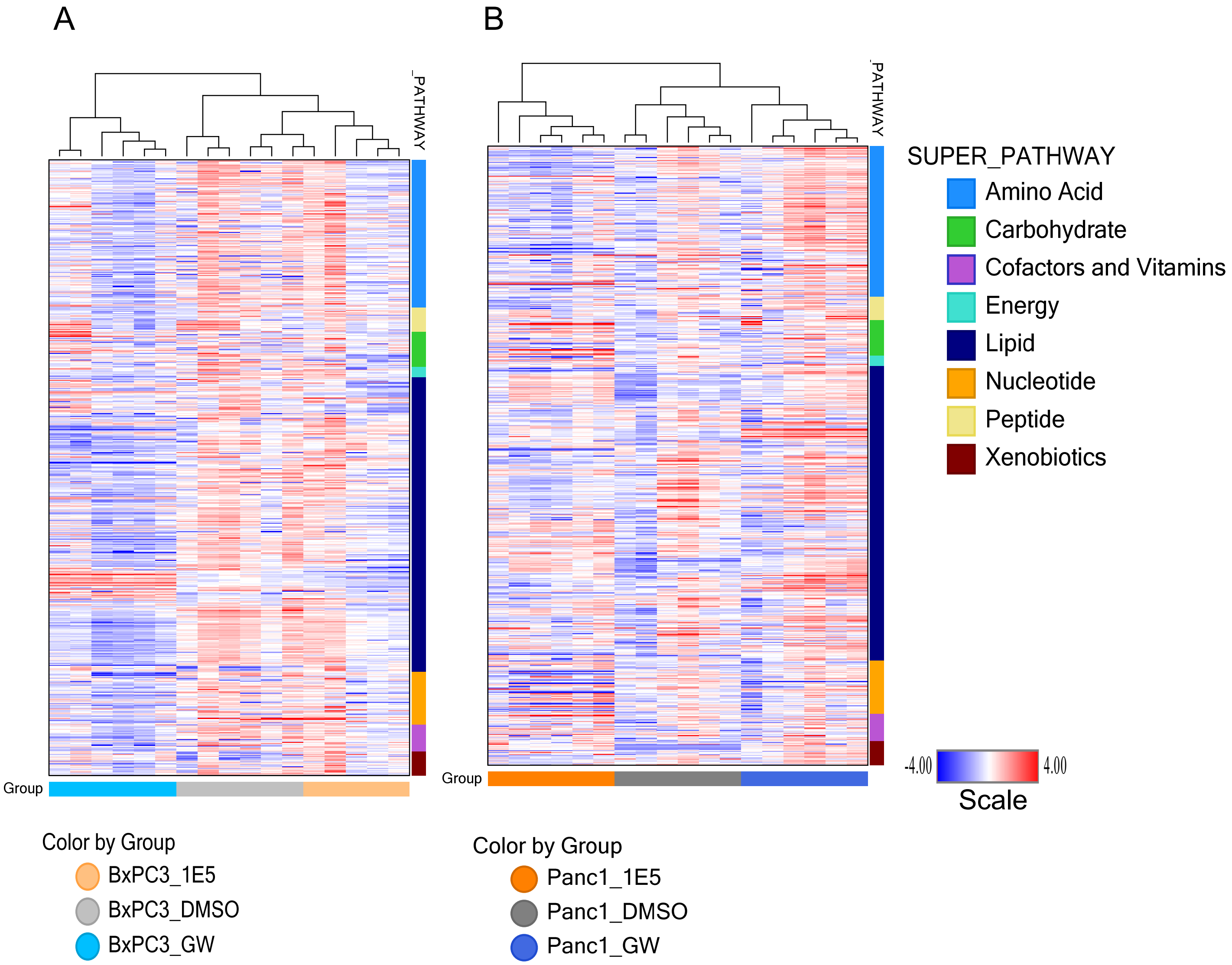

Supplementary Figure S1

A

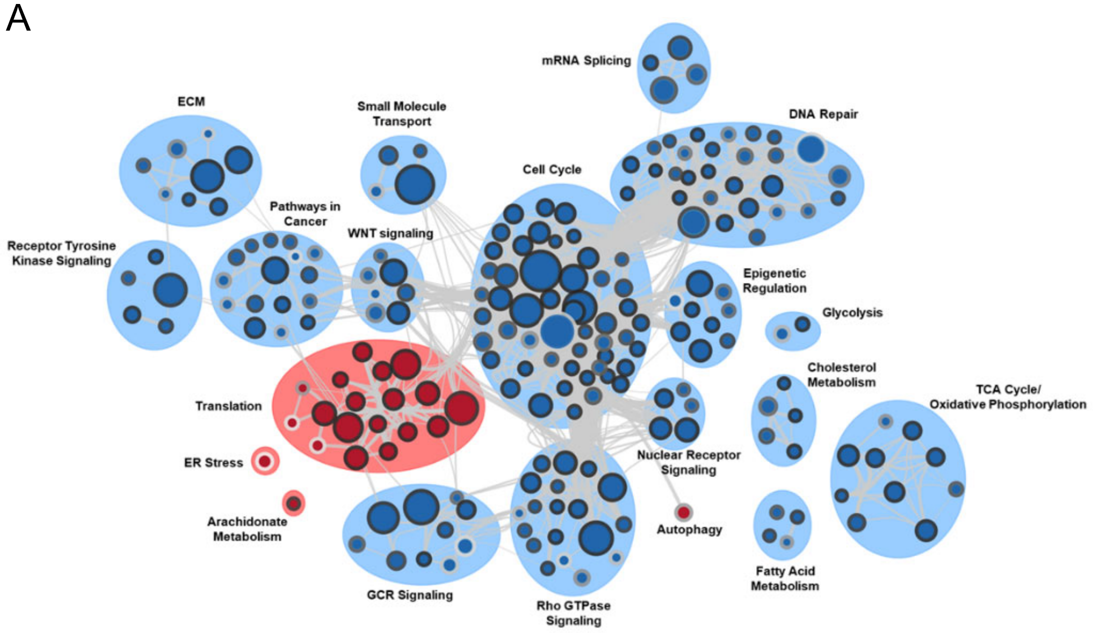

B

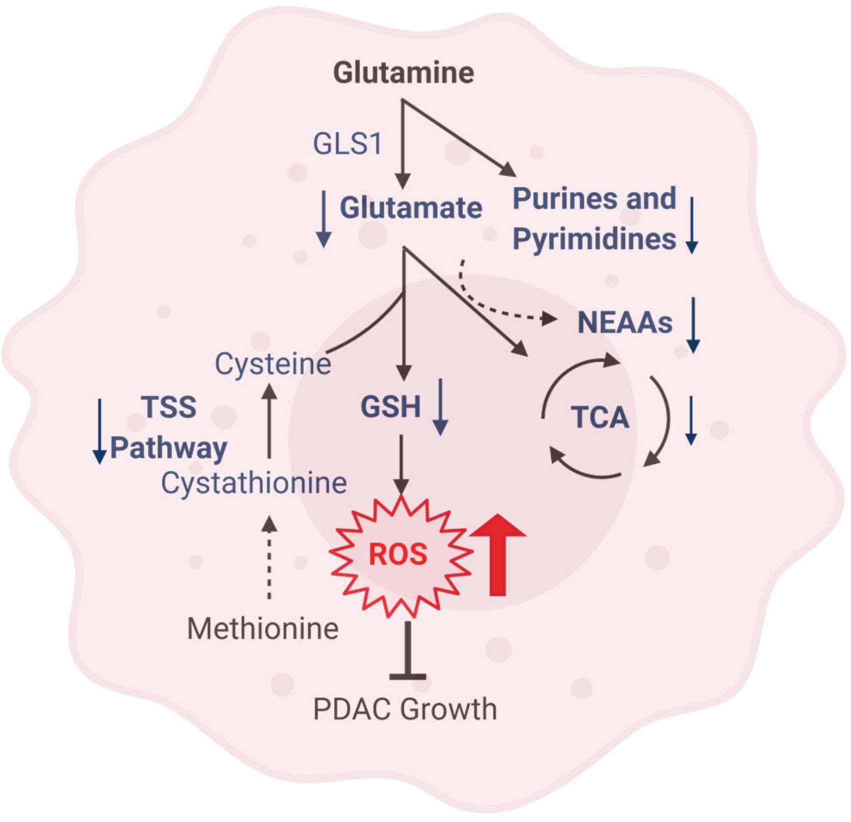

Supplementary Figure S2

**Table S1: Summary of differentially regulated metabolites identified in metabolomics study**				
**Treatment/DMSO**	**GW3965**		**1E5**	
**Cell Line**	**Upregulated**	**Downregulated**	**Upregulated**	**Downregulated**
**BxPC-3**	83	367	97	142
**PANC-1**	216	81	177	187

**Table S2: Joint pathway analysis of PANC-1 cells upon 1E5 treatment**				
**Pathway Name**	**Total Metabolites**	**Raw p-value**	**FDR**	**Impact**
Purine metabolism	225	2.19E-75	7.24E-73	4.5792
Pyrimidine metabolism	122	3.42E-51	5.66E-49	4.1318
Cysteine and methionine metabolism	112	2.04E-48	2.25E-46	2.2718
Alanine, aspartate and glutamate metabolism	64	1.63E-47	1.35E-45	4.2623
Central carbon metabolism in cancer	106	9.62E-38	6.37E-36	0.13274
Glutathione metabolism	94	1.55E-31	8.58E-30	2.5362
Citrate cycle (TCA cycle)	50	2.29E-30	1.08E-28	3.1818
Glycolysis or Gluconeogenesis	99	1.23E-25	5.10E-24	2.4308
Glycine, serine and threonine metabolism	90	3.14E-17	1.04E-15	1.6588
Arginine biosynthesis	44	2.07E-16	6.23E-15	1.7143
Pyruvate metabolism	70	4.87E-16	1.34E-14	1.7925

Table S3: Joint pathway analysis of BxPC-3 cells upon 1E5 treatment				
**Pathway Name**	**Total Metabolites**	**Raw p-value**	**FDR**	**Impact**
Aminoacyl-tRNA biosynthesis	74	2.42E-06	0.00020364	0.24658
Arginine biosynthesis	27	8.51E-06	0.00035728	0.88462
Alanine, aspartate and glutamate metabolism	61	1.53E-05	0.00042962	0.95
Purine metabolism	166	8.23E-05	0.0013949	0.95758
Valine, leucine and isoleucine biosynthesis	12	8.30E-05	0.0013949	0.72727
Histidine metabolism	32	0.00028326	0.0038105	0.64516
Arginine and proline metabolism	78	0.00031754	0.0038105	0.5974
Pyrimidine metabolism	99	0.0005203	0.0054631	0.91837
Citrate cycle (TCA cycle)	42	0.0023964	0.022366	0.97561
beta-Alanine metabolism	44	0.0033599	0.028223	0.7907
Taurine and hypotaurine metabolism	16	0.0041873	0.031976	0.73333
